# Supplementary material for: FERN – a Java framework for stochastic simulation and evaluation of reaction networks
Source: BMC Bioinformatics. 2008 Aug 29;9:356. doi: 10.1186/1471-2105-9-356 (PMC2553347; doi:10.1186/1471-2105-9-356)
Supplement: Additional file 1 — FERN distribution, Version 1.3. This archive contains the FERN source code and binaries as well as documentation and example models in FernML and SBML. [file 1471-2105-9-356-S1.zip › fern/doc/javadoc/fern/simulation/controller/SimulationController.html]

SimulationController


---


|  |  |  |
| --- | --- | --- |
| **Overview** \| **Package** \| **Class** \| **Use** \| **Tree** \| **Deprecated** \| **Index** \| **Help** | |  |
| **PREV CLASS**   NEXT CLASS | **FRAMES**    **NO FRAMES**     **All Classes** |  |
| SUMMARY: NESTED \| FIELD \| CONSTR \| METHOD | DETAIL: FIELD \| CONSTR \| METHOD |  |


---


## fern.simulation.controller Interface SimulationController

**All Known Implementing Classes:**: AmountAtMomentObserver, AndController, DefaultController, OrController

---

``` public interface SimulationController ```

`Simulator`s are controlled by implementing classes. Each simulation step
the `goOn` method is called to check whether to go on or not.

**Author:**
:   Florian Erhard

---

| **Method Summary** | |
| --- | --- |
| `boolean` | `goOn(Simulator sim)`             Returns whether or not to go on with the given simulation |

**Method Detail**

### goOn

```
boolean goOn(Simulator sim)
```

:   Returns whether or not to go on with the given simulation

    :   **Parameters:**: `sim` - simulation **Returns:**: whether or not to go on


---


|  |  |  |
| --- | --- | --- |
| **Overview** \| **Package** \| **Class** \| **Use** \| **Tree** \| **Deprecated** \| **Index** \| **Help** | |  |
| **PREV CLASS**   NEXT CLASS | **FRAMES**    **NO FRAMES**     **All Classes** |  |
| SUMMARY: NESTED \| FIELD \| CONSTR \| METHOD | DETAIL: FIELD \| CONSTR \| METHOD |  |


---
